# Supplementary material for: Surface Microbiome Profiling of Dental Elevators Using Third‐Generation Sequencing: Implications for Infection Control in Dental Practice
Source: Microbiologyopen. 2025 Dec 8;14(6):e70178. doi: 10.1002/mbo3.70178 (PMC12685768; doi:10.1002/mbo3.70178)
Supplement: Supplementary file 1 — customer_backup. [file MBO3-14-e70178-s001.zip › customer_backup/customer_backup/function_analysis/KEGG/allsample/K2species/allsample.reabundance_Class2Class.txt.pdf]

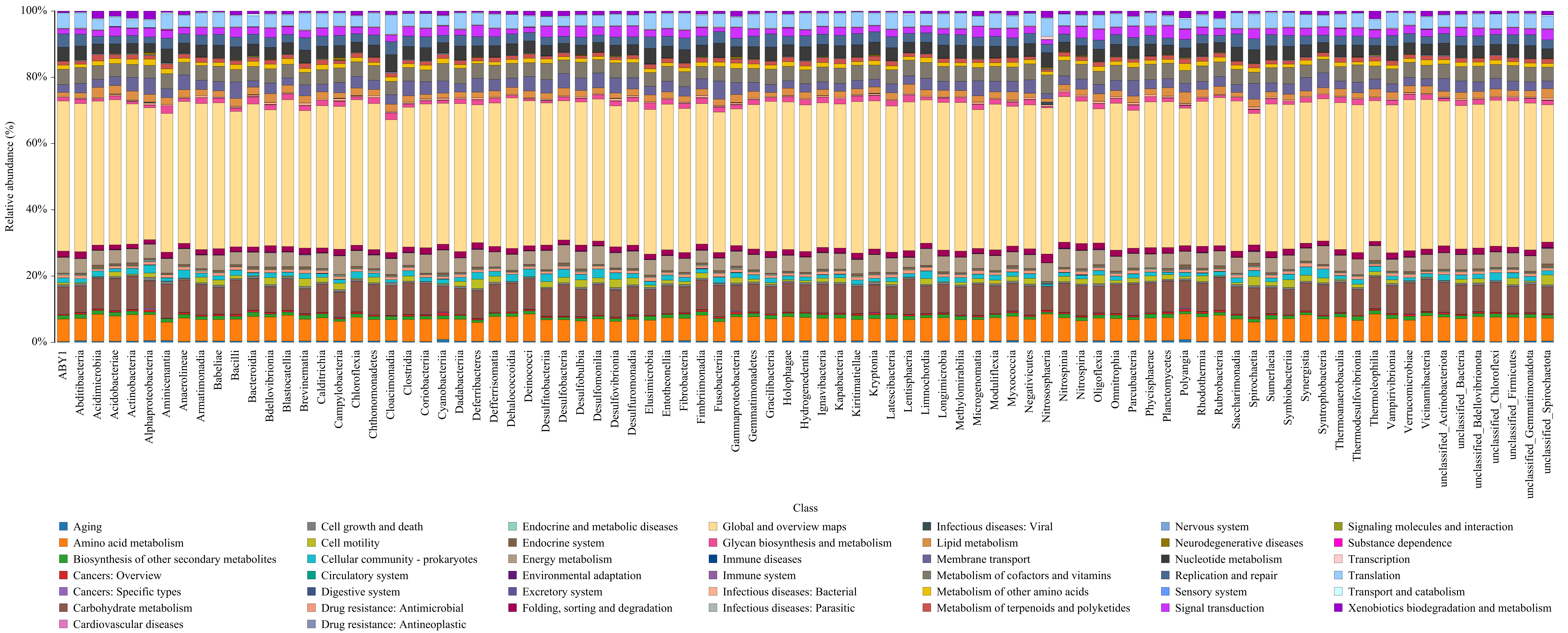
100%
80%
60%
40%
20%
0%
Relative abundance (%)
ABY1
Abditibacteria
Acidimicrobiia
Acidobacteriae
Actinobacteria
Alphaproteobacteria
Aminicenantia
Anaerolineae
Armatimonadia
Babeliae
Bacilli
Bacteroidia
Bdellovibrionia
Blastocatellia
Brevinematia
Caldatribacteriia
Campylobacteria
Chloroflexia
Chthonomonadetes
Cloacimonadia
Clostridia
Coriobacteriia
Cyanobacteriia
Dadabacteriia
Deferribacteres
Deferrisomatia
Dehalococcoidia
Deinococci
Desulfitobacteriia
Desulfobacteria
Desulfobulbia
Desulfomonilia
Desulfovibrionia
Desulfuromonadia
Elusimicrobia
Entotheonellia
Fibrobacteria
Fimbriimonadia
Fusobacteriia
Gammaproteobacteria
Gemmatimonadetes
Gracilibacteria
Holophagae
Hydrogenedentia
Ignavibacteria
Kapabacteria
Kiritimatiellae
Kryptonia
Latescibacteria
Lentisphaeria
Limnochordia
Longimicrobia
Methylomirabilia
Microgenomatia
Moduliflexia
Myxococcia
Negativicutes
Nitrososphaeria
Nitrospinia
Nitrospiria
Oligoflexia
Omnitrophia
Parcubacteria
Phycisphaerae
Planctomycetes
Polyangia
Rhodothermia
Rubrobacteria
Saccharimonadia
Spirochaetia
Sumerlaeia
Symbiobacteriia
Synergistia
Syntrophobacteria
Thermoanaerobaculia
Thermodesulfovibrionia
Thermoleophilia
Vampirivibrionia
Verrucomicrobiae
Vicinamibacteria
unclassified_Actinobacteriota
unclassified_Bacteria
unclassified_Bdellovibrionota
unclassified_Chloroflexi
unclassified_Firmicutes
unclassified_Gemmatimonadota
unclassified_Spirochaetota
Class
Aging
Amino acid metabolism
Biosynthesis of other secondary metabolites
Cancers: Overview
Cancers: Specific types
Carbohydrate metabolism
Cardiovascular diseases
Cell growth and death
Cell motility
Cellular community - prokaryotes
Circulatory system
Digestive system
Drug resistance: Antimicrobial
Drug resistance: Antineoplastic
Endocrine and metabolic diseases
Endocrine system
Energy metabolism
Environmental adaptation
Excretory system
Folding, sorting and degradation
Global and overview maps
Glycan biosynthesis and metabolism
Immune diseases
Immune system
Infectious diseases: Bacterial
Infectious diseases: Parasitic
Infectious diseases: Viral
Lipid metabolism
Membrane transport
Metabolism of cofactors and vitamins
Metabolism of other amino acids
Metabolism of terpenoids and polyketides
Nervous system
Neurodegenerative diseases
Nucleotide metabolism
Replication and repair
Sensory system
Signal transduction
Signaling molecules and interaction
Substance dependence
Transcription
Translation
Transport and catabolism
Xenobiotics biodegradation and metabolism
